# Randomized, double-blind, placebo-controlled study of oral duloxetine in prevention of acute pain syndrome in breast cancer patients receiving paclitaxel (DOPA study)

**DOI:** 10.1186/s13063-026-09673-9

**Published:** 2026-04-18

**Authors:** Jahnavi Banotra, Vrajesh Veenadhar Shetty, Sameer Bakhshi, Achal Kumar Srivastava, Sudeep Gupta, Akash Kumar, Prabhat Bhargava, Bharath Gangadhar, Payal Vasudeva, Kriti Pallavi, Kamesh Bhati, Shlok Singh, Neha Verma, Mohit Kumar Divakar, Hemavathi Baskarane, Atul Batra

**Affiliations:** 1 All India Institute of Medical Sciences, New Delhi, 110029 India; 2 Department of Medical Oncology, Tata Memorial Centre, Mumbai, India

**Keywords:** Paclitaxel, Acute pain syndrome, Duloxetine, Myalgia, Breast cancer

## Abstract

**Background:**

Acute pain syndrome (APS) is a common side effect of paclitaxel therapy. To date, there is no standard of care to prevent APS in patients receiving paclitaxel. Through this study, we aim to assess whether oral duloxetine reduces the incidence of paclitaxel-induced APS (P-APS) as compared to placebo.

**Methods:**

This is a multi-centric, randomized (1:1), double-blind, placebo-controlled, parallel-group superiority trial. A total of 204 patients with breast cancer planned to receive paclitaxel will be randomly assigned to receive either oral duloxetine or a matched placebo for 7 days after paclitaxel infusions for 4 cycles. The primary objective of the study is to compare the proportion of patients who develop P-APS in two groups. Key secondary objectives are to compare the quality of life (using the FACT-B scale), the incidence of peripheral neuropathy, the safety profile, and adherence with duloxetine. Patient-reported outcomes for P-APS and neuropathy will be assessed at the end of each cycle using BPI-SF and EORTC-CIPN20 questionnaires, respectively.

**Discussion:**

The DOPA study is designed to evaluate whether oral duloxetine can reduce the occurrence of APS in patients receiving paclitaxel chemotherapy for breast cancer. Limited trials have assessed P-APS prevention using etoricoxib, dexamethasone, and pregabalin, but no pharmacological measure is effective. If the trial successfully meets its primary endpoint, oral duloxetine could become the new standard of care for preventing paclitaxel-induced APS.

**Trial registration:**

Clinical Trials Registry of India – CTRI/2024/10/075636. Registered on 22 October 2024.

## Administrative information

Note: This protocol uses curly‑bracketed numbers to denote SPIRIT checklist items, with the sequence rearranged to group related topics together.

**Table:** 

Title {1}	Randomized, double-blind, placebo-controlled study of oral duloxetine in prevention of acute pain syndrome in breast cancer patients receiving paclitaxel
Trial registration {2a and 2b}	Clinical Trials Registry of India (CTRI/2024/10/075636)
Protocol version {3}	Protocol version 1.1 August 22nd, 2025
Funding {4}	Funding for this trial was provided by an intramural grant from the All India Institute of Medical Sciences, New Delhi, India (No.F.5–59/IRG/2010/RS).
Author details {5a}	Jahnavi Banotra^1$^Vrajesh Veenadhar Shetty^1$^, Sameer Bakhshi^1^ Achal Kumar Srivastava^1^, Sudeep Gupta^2^,,Akash Kumar^1^,Prabhat Bhargava^2^, Bharath Gangadhar^1^,Payal Vasudeva^1^, Kriti Pallavi^1^, Kamesh Bhati^1^, Shlok Singh^1^, Neha Verma^1^, Mohit Kumar Divakar^1^, Hemavathi Baskarane^1#^, Atul Batra^1#^ Author details: 1 All India Institute of Medical Sciences, New Delhi 110,029, India. 2 Department of Medical Oncology, Tata Memorial Centre, Mumbai, India.
Name and contact information for the trial sponsor {5b}	Dr Atul Batra, Additional Professor, Department of Medical Oncology, Dr BR Ambedkar Institute Rotary Cancer Hospital, All India Institute of Medical Sciences, New Delhi, India
Role of sponsor {5c}	This is an investigator-initiated multicentric study, with the lead center being the All India Institute of Medical Sciences (AIIMS), New Delhi. The principal investigator is employed at AIIMS, New Delhi (the study sponsor), which is a tertiary-care, academic teaching hospital and medical school. The hospital provides administrative, logistical, and other support for this study.

## Introduction

### Background and rationale {6a}

Paclitaxel, a widely used chemotherapeutic agent in the treatment of various malignancies, inhibits microtubule depolymerization, thereby disrupting cancer cell division [1]. It is commonly used in several clinical settings in breast cancer, including adjuvant, neoadjuvant, and palliative chemotherapy regimens [2]. Despite its established efficacy, the clinical application of paclitaxel is frequently constrained by the development of paclitaxel-associated acute pain syndrome (P‑APS), a debilitating adverse effect characterized by severe neuropathic pain, myalgias, and arthralgias [3]. Reported incidence rates of P‑APS vary considerably across studies, ranging from 20 to 88% [3, 4]. Although not life-threatening, P-APS compromises patients’ quality of life [5].

Clinically, P‑APS most commonly presents as a diffuse, aching discomfort localized to the lower extremities, hips, and lumbar region, though widespread involvement can occur [6]. The symptoms of P‑APS generally reach peak intensity around the third day post-chemotherapy, with 20% reporting pain scores exceeding 4/10 after the initial dose [4]. Elevated pain scores during the first treatment cycle are predictive of a higher likelihood of chronic neuropathy development [3, 7]. As P-APS typically occurs early during treatment and may affect treatment tolerance and patients quality of life, identifying effective preventive strategies remains an important unmet clinical need.

The exact pathophysiology of P-APS remains incompletely understood, but it is believed to involve nerve inflammation, injury, and nociceptive sensation [4, 6]. Dougherty et al. demonstrated a marked impairment of large myelinated A‑beta fibers, with relatively minor effects on small myelinated A‑delta fibers [8].

Currently, no effective prophylactic agents for P-APS exist. Both non‑opioid and opioid analgesics are used for the relief of symptoms. Several strategies, including Shakuyaku-Kanzo-To (a Japanese herb), antihistamines, corticosteroids, analgesics, metformin, and amifostine, have been studied for their neuroprotective effects and potential in pain prevention [9]. Dexamethasone is associated with a decrease in the severity of P-APS, but no difference in incidence [10]. Etoricoxib, in phase II studies, demonstrated a reduction in both the overall incidence and severity of docetaxel-induced acute pain syndrome [11]. Most other pharmacological interventions have failed to prevent P-APS [12–14]. Given the limited success of current management strategies, there is an unmet need for an effective prophylactic intervention of P-APS.

Duloxetine, a selective serotonin and norepinephrine reuptake inhibitor (SNRI), has demonstrated efficacy in managing neuropathic pain associated with diabetic peripheral neuropathy and fibromyalgia [15–17]. Duloxetine’s dual inhibition of serotonin and norepinephrine reuptake is believed to modulate neurotransmitter levels and nociceptive pathways [18, 19]. Exposure to paclitaxel leads to central sensitization, amplifying pain perception [20]. Duloxetine’s modulatory effects on central pain pathways might help in interrupting this process, thus preventing P-APS progression and improving pain outcomes. Furthermore, duloxetine possesses a well‑established safety profile and is generally well‑tolerated [19]. Accordingly, we designed this study to evaluate its efficacy and safety in the prevention of P‑APS among patients with breast cancer.

### Objectives {7}

We hypothesize that prophylactic duloxetine, given 30 mg once daily for 7 days after each cycle of paclitaxel administration, will reduce the incidence of P-APS by 20%, compared with placebo (30 vs 50%, respectively).

The primary objective of this study is as follows:

1. To compare the effect of oral duloxetine with placebo in preventing P-APS.

The secondary objectives of this study are to compare the effect of oral duloxetine with placebo on:

1. The proportion of patients who develop peripheral neuropathy in each arm, assessed clinically, using the EORTC-CIPN 20 questionnaire, and objectively via nerve conduction study.
2. Patients’ quality of life (using the FACT-B scale) in the two arms.
3. Adherence rates
4. Safety profile of duloxetine

## Methods: participants, interventions, and outcomes

### Trial design {8}

This study is a multi-center, parallel-group, double-blind, 1:1 randomized, placebo-controlled superiority trial. It includes two centers: AIIMS, New Delhi, and Tata Memorial Centre, Mumbai.

### Study setting {9}

The study will be conducted in the outpatient breast cancer clinics at the Dr. BR Ambedkar Institute Rotary Cancer Hospital (IRCH), AIIMS, New Delhi, and the Tata Memorial Centre (TMC), Mumbai. IRCH is the dedicated cancer facility of AIIMS, New Delhi, a central government-funded tertiary care hospital and academic center. It serves a vast catchment area comprising Delhi and neighboring states, with a combined population exceeding 20 million, and registers more than 15,000 new cancer patients annually. TMC is located in Mumbai and caters to the western and northern states in India, and registers and treats over 60,000 patients with cancer annually.

### Eligibility criteria {10}

Patients will be eligible for participation if they meet all of the following inclusion criteria:

1. Breast cancer patients (males or females) of age ≥ 18 years.
2. Diagnosis of breast cancer confirmed by either biopsy or surgical resection specimen.
3. Scheduled to receive paclitaxel at a dose of 175 mg/m^2^, administered either every 2 weeks or every 3 weeks, with or without carboplatin, in the neoadjuvant or adjuvant setting.
4. Eastern Cooperative Oncology Group (ECOG) performance status 0–2.
5. Study treatment planned and feasible to commence within 14 days of randomization.
6. Willing and able to adhere to all study requirements, including oral administration of duloxetine, treatment schedule, and completion of required assessments.

Patients who meet any of the following criteria will be excluded from participation in the trial:

1. Patients planned to receive paclitaxel in combination with carboplatin for six cycles, including those receiving this regimen in breast cancer (neoadjuvant, adjuvant, or metastatic setting) or in ovarian cancer.
2. Pre-existing chronic pain or peripheral neuropathy assessed clinically and evaluated using the Brief Pain Inventory (BPI-SF) and the CIPN20 questionnaire, respectively.
3. Uncontrolled diabetes (HbA1c > 8gm/dl).
4. Pregnant women.
5. Previously received paclitaxel or chemotherapeutic agents causing neuropathy.
6. Any prevailing medical or psychiatric condition that, in the investigator’s judgment, may jeopardize or compromise the patient’s ability to participate in the study.
7. Currently taking steroids and pain medications (NSAIDS, opioids, gabapentin, pregabalin and SSRIs).
8. History of known allergy or hypersensitivity to either study drugs or their components, as specified in the protocol.
9. Concomitant use of CYP3A4 enzyme inducers (e.g., phenytoin, carbamazepine, rifampicin, barbiturates).
10. Patients with severe renal impairment (creatinine clearance < 30 mL/min), end‑stage renal disease, or hepatic impairment (defined as transaminase levels exceeding three times the upper limit of normal or bilirubin levels exceeding twice the upper limit of normal).

### Informed consent {26a}

Written informed consent will be obtained by the study investigator from each participant prior to enrollment in the clinical trial. During the consent process, the potential risks and benefits of study participation will be explained in a language understandable to the patient (Hindi or English or Marathi). Participation will be entirely voluntary, with consent given freely and without any form of coercion.

### Additional consent provisions for collection and use of participant data and biological specimens {26b}

Not applicable.

### Interventions

#### Explanation for the choice of comparators {6b}

Patients will be randomly assigned to one of two study arms: the intervention arm, receiving 30 mg duloxetine, or the control arm, receiving a matched placebo. Placebo was selected as the comparator because no established standard‑of‑care intervention currently exists for the prevention of P‑APS.

#### Intervention description {11a}

The intervention in this trial is 30 mg of duloxetine administered orally at bedtime for 7 days, starting from day 1 to 7 of each of the four paclitaxel cycles. The placebo will be packed in identical, unlabeled bottles by INTAS Pharmaceuticals Ltd. Bottles will then be labeled by an independent research staff using the alphanumeric codes (e.g., AA1) generated for each randomization number, and this personnel will have no role in the conduct of this study to maintain blinding. Each participant will receive a bottle containing 30 tablets, sufficient to cover the dosing required for four cycles, and the remaining tablets and bottle will be collected at the end of the study.

### Criteria for discontinuation {11b}

The study treatment will be discontinued in case the patient meets any of the following criteria:

- Permanent discontinuation of paclitaxel by treating physician due to unacceptable toxicity or progression of underlying cancer.
- Completion of four cycles of paclitaxel therapy.
- Decision by the investigator that continuing treatment is not in the patient’s best interest.
- Occurrence of an event affecting patient safety, e.g., pregnancy or psychiatric illness (any exclusion criteria).
- Requirement for a concomitant treatment that is not permitted.
- Non-compliance with the protocol, for example, repeated failure to attend scheduled study assessments. If a patient misses scheduled assessments, the investigator must identify and document the reasons and circumstances in detail within the medical records and Case Report Form (CRF).
- Patient refusal to continue study treatment or withdrawal of consent to participate in the study.

All reasons for treatment discontinuation will be recorded in the subject’s medical records.

### Strategies to improve adherence to interventions {11c}

**Fig. 1:**
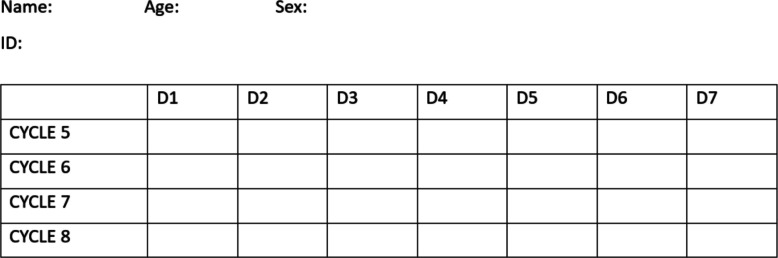
Self-reporting sheet

At the time of randomization, patients will receive a self-reporting adherence sheet, which they will mark after taking the prescribed medication. This will be checked, and the remaining tablets will be counted at every visit. The patients will be counseled appropriately by the study nurse if significant non-adherence is determined (Fig. 1).

### Relevant concomitant care permitted or prohibited during the trial {11d}

Following consultation with the clinical trial coordinator, patients will be permitted to receive standard-of-care treatment at the discretion of the treating physician. The use of supportive care medications, including but not limited to antiemetics, anti-diarrheals, and antibiotics, will be allowed. Administration of monoamine oxidase inhibitors (MAOIs) is strictly prohibited. Oral nonsteroidal anti-inflammatory drugs (NSAIDs) and aspirin should be strongly avoided throughout the study. In cases of clinically significant pain, oral paracetamol, NSAIDs, or tramadol may be administered as rescue therapy. All instances of rescue medication use must be documented in the case record form at the patient’s subsequent study visit.

### Ancillary and post-trial care {30}

Since no established standard of care exists for the prevention of P-APS, post-trial management will be determined at the discretion of the treating oncologist. No further supply of duloxetine will be provided beyond the duration of the study.

### Outcomes {12}

The primary outcome of the study is the proportion of patients who develop APS, defined as BPI-SF > 0, at any time during the four cycles of paclitaxel treatment, compared between the duloxetine and placebo arms. APS will be assessed using the BPI-SF scale from day 1 to 7 of each cycle, with severe APS defined as a BPI-SF score > 5. The primary and secondary outcomes of the study are described in Table 1.

**Table 1:** Primary and secondary outcomes of the study

Outcome	Description
Primary	Proportion of patients who develop acute pain syndrome (APS) during treatment, defined as the incidence of pain (BPI-SF score > 0)
Secondary	Proportion of patients who develop severe APS, defined as BPI-SF score > 5
	Incidence of peripheral neuropathy assessed using the CIPN-20 questionnaire
	Quality of life using the FACT-B questionnaire
	Compliance with oral duloxetine
	Adverse events graded according to NCI CTCAE version 5.0

### Participant timeline {13}

Patients will be followed up for a period of 3 months after completion of the fourth cycle of paclitaxel, with the objective of evaluating peripheral neuropathy. Baseline demographic characteristics will be documented prior to initiation of therapy. Subsequently, at the end of each treatment cycle, data collection will include clinical assessments, relevant outcome measures, treatment-related parameters, and any adverse events. These data will be retrieved from hospital medical records, patient-reported questionnaires, and relevant laboratory investigations.

#### Timeline

**Table:** 

	Screening	Baseline	On treatment	Completion of chemotherapy	End of assessment
	Within 14 days prior to registration	Within 3 days prior to day 1 of cycle 1	Cycle 2–4	Within three days at the end of cycle 4	2–3 months after the end of cycle 4
Informed consent	X				
Clinic assessment	X	X	X	X	X
Concomitant medications		X	X	X	
Adverse events			X	X	
P-APS assessment: clinical		X (Day1–7)	X	X	
Paclitaxel dose/schedule		X	X	X	
Quality of life assessment (FACT-B)		X	X	X	X
Neuropathy (EORTC—CIPN20)	X	X	X	X	X
Nerve Conduction Study (NCS)	X				X
Patient adherence (self-reporting sheets)			X	X	

### Sample size {14}

With a total of 204 participants (102 per arm), this trial is designed with at least 80% power to detect a 20% absolute difference in the incidence of P-APS between the two study groups, based on a chi-squared test with a two-sided significance level of 0.05. It is assumed that approximately 50% of patients in the placebo group will develop P-APS, whereas the duloxetine group is expected to show a reduction to 30%. Participants will be randomized in a 1:1 ratio to receive either duloxetine or placebo, beginning from the first cycle of paclitaxel.

### Recruitment {15}

Patients will be recruited independently at each participating center. Recruitment will be conducted through the outpatient breast cancer clinics at the Dr. B. R. Ambedkar Institute Rotary Cancer Hospital, All India Institute of Medical Sciences (AIIMS), New Delhi, and the Tata Memorial Centre (TMC), Mumbai. The enrollment period is expected to be completed within 1 year from study initiation.

## Methods: assignment of interventions

### Randomization

#### Sequence generation {16a}

Participants will be assigned to the duloxetine or placebo arm in a 1:1 ratio using a computer-generated permuted block randomization sequence with variable block sizes, in order to maintain allocation concealment and ensure balanced distribution across arms. The randomization sequence will be prepared and maintained by an independent statistician who has no involvement in patient recruitment, clinical management, outcome assessments, or any other aspect of the trial. Stratified randomization based on concurrent carboplatin (combination vs monotherapy) will be used in the study. Randomization will be done centrally at AIIMS, New Delhi.

#### Allocation concealment mechanism {16b}

Allocation will be carried out through the use of sealed, opaque envelopes, thereby maintaining allocation concealment, and drugs will be dispensed by research personnel not involved in further study conduct.

#### Implementation {16c}

Participants will be screened in the outpatient clinics before the first cycle of paclitaxel. After screening, eligible patients will be randomized centrally.

## Assignment of interventions: blinding

This study is designed as a double-blind randomized controlled trial. Both participants and investigators will remain blinded to treatment assignments throughout the trial.

### Process of blinding {17a}

This trial is a double-blind study. To maintain blinding, duloxetine and placebo tablets, containers, and labels will be identical, ensuring blinding of investigators and participants. Randomization and drug dispensing will be done by independent research staff not involved in further conduct of the study.

### Procedure for unblinding {17b}

Blinding will only be broken in the event of a medical emergency, when knowledge of the study drug is crucial for the participant’s immediate clinical management. In such cases, the investigator will unblind the treatment and inform the Institutional Ethics Committee.

### Methods: data collection, management, and analysis

#### Plans for assessment and collection of outcomes {18a}

Research staff will collect the data at baseline and the end of each chemotherapy cycle. Printed copies of case record forms will be used for the same. The EORTC CIPN 20 and FACT-B questionnaires will be completed in the clinic alongside clinical assessments. BPI-SF forms will be provided to patients at each visit for self-recording from day 1 to day 7. All data will be collated and securely maintained.

#### Plans to promote patient retention and complete follow-up {18b}

Participants will be assessed prior to the initiation of cycle 1 of paclitaxel and before the start of each subsequent cycle, approximately 2 to 3 days before drug administration. A follow-up visit will be scheduled 8 to 12 weeks after completion of treatment to assess neuropathy using a nerve conduction study. They will be contacted by telephone in the event of a missed appointment.

### Data management {19}

All study data will be manually entered and coded by designated research staff, and subsequently stored in secure, password-protected folders. Access to these data will be strictly restricted to authorized personnel only.

### Confidentiality {27}

Consent forms containing participants’ identifying information will be securely stored at the study site and accessible only to authorized personnel. Wherever possible, personal identifiers will be removed, and participants will instead be referenced by a unique study identification number. The case record forms will not contain any identifying data.

### Statistical methods

#### Plans for collection, laboratory evaluation, and storage of biological specimens for genetic or molecular analysis in this trial/future use {33}

Not applicable.

#### Statistical methods for primary and secondary outcomes {20a}

The primary endpoint will be compared between the two study groups using a chi-squared test, with the risk difference reported alongside a 95% confidence interval. A two-sided significance level of 0.05 will be applied.

Patient demographics, clinical and treatment characteristics, and other study outcomes will be summarized within each randomized group and overall. Continuous variables will be described using mean, standard deviation, and range, or median with interquartile range and range, as appropriate. Categorical variables will be summarized as frequencies and percentages. Time-to-event variables will be analyzed using the Kaplan–Meier method.

Comparisons between groups will be performed using standard statistical tests: two-sample *t*-tests or non-parametric equivalents for continuous outcomes, chi-squared tests for categorical outcomes, and the log-rank test for time-to-event outcomes. Effect sizes with corresponding 95% confidence intervals will be reported where applicable. All analyses will be conducted according to the intention-to-treat principle, whereby all randomized participants will be analyzed in the treatment group to which they were allocated, irrespective of the treatment actually received.

#### Interim analyses {21b}

No interim analyses are planned for this trial, given the minimal safety concerns associated with oral duloxetine.

#### Methods for additional analyses (e.g., subgroup analyses) {20b}

Exploratory analyses to identify predictors of outcomes and to conduct adjusted assessments will be performed using regression models, including logistic regression for binary outcomes and linear regression for continuous outcomes.

#### Methods in analysis to handle protocol non-adherence and any statistical methods to handle missing data {20c}

Missing data will be evaluated to determine the underlying mechanism. The assumption of data missing completely at random (MCAR) will be formally assessed using Little’s test. If data are deemed missing at random (MAR), multiple imputation methods will be applied. For data not considered MAR, appropriate sensitivity analyses (e.g., worst-case/best-case scenarios) will be performed to assess the potential impact of missingness on study conclusions.

Sensitivity analyses will be undertaken using the per-protocol population to evaluate the robustness of findings in participants with high adherence to the study protocol.

#### Plans to give access to the full protocol, participant-level data, and statistical code {31c}

Not applicable.

## Oversight and monitoring

### Composition of the coordinating center and trial steering committee {5d}

An independent Data Safety Monitoring Board (DSMB) will be established, comprising experts from various fields. The DSMB will be informed of all serious adverse events (SAEs) and any grade 3 or 4 non-hematological toxicities within 72 h of notification by the principal investigator. The DSMB will convene every 6 months to review all adverse event data and perform causality assessments. Based on the information provided, the DSMB will offer timely recommendations to the investigators.

All support staff, clinicians, investigators, and trial coordinators will undergo regular quality assurance training related to trial procedures. This training will occur at frequent intervals.

Key quality metrics will be tracked, reported, and reviewed throughout the trial. All protocol deviations will be documented and assessed for severity. Any major protocol deviations will be reported to the principal investigator and DSMB, followed by corrective and preventive actions to prevent recurrence.

The trial nurse shall screen the eligible patients, counsel, and explain about the benefits and risks involved. The PI shall review and finalize enrollment and obtain consent. Randomization will be blinded, done by trial coordinator. Ensuring uninterrupted supply of drugs and other materials shall be ensured by the coordinator. The PI and trial nurse shall monitor for toxicity. The coinvestigators shall ensure that the trial proceeds smoothly.

### Composition of the data monitoring committee, its role, and reporting structure {21a}

A formal data monitoring committee will not be constituted for this study, as the safety risks associated with oral duloxetine are considered minimal. Oversight of safety will instead be provided by the Trial Steering Committee, which will be responsible for reviewing all serious adverse events (SAEs). Any SAEs identified will be reported to the Institute Ethics Subcommittee for Monitoring of Adverse Events in Clinical Trials at the study site per institutional and regulatory requirements.

### Adverse event reporting and harms {22}

Adverse events will be collected and graded according to the National Cancer Institute’s Common Terminology Criteria for Adverse Events (NCI-CTCAE), version 5.0, at the end of each treatment cycle. Serious adverse reactions (SARs) occurring during the study will be reported promptly to the Institutional Ethics Subcommittee for Monitoring of Adverse Events in Clinical Trials, in compliance with applicable reporting guidelines.

### Frequency and procedures for monitoring trial conduct {23}

Study data will be available for review by the Institutional Ethics Committee (IEC), if required. In addition, this study may be subject to audit or inspection by the IEC and by representatives of the Drug Controller General of India (DCGI).

### Plans for communicating important protocol amendments to relevant parties (e.g., trial participants, ethical committees) {25}

Any amendments to the study protocol will be reported promptly to the Institutional Ethics Committee and the Clinical Trials Registry of India (CTRI). All participating centers will also be notified in a timely manner.

## Dissemination plans {31a}

Study results will also be presented at major scientific conferences, and a summary of findings will be made available to all study participants. Furthermore, detailed study findings will be submitted for publication in a peer-reviewed scientific journal, with appropriate authorship and credit given to all investigators and research staff who contributed to the work. All authors will review and approve the manuscript before submission, in accordance with internationally recognized standards.

### Conclusions

Paclitaxel, a commonly used chemotherapeutic agent in various malignancies and an integral part of neoadjuvant and adjuvant regimens in breast cancer, is associated with the debilitating adverse effect of acute pain syndrome, occurring at various frequency and severity, along with long-term neurotoxicity. Currently, no effective strategies exist for the prevention of APS. The DOPA study is a randomized, double-blind, placebo-controlled, parallel-group phase III superiority trial designed to evaluate the efficacy of duloxetine in preventing P-APS.

## Discussion

Not applicable.

### Trial status

The first participant was enrolled on January 8, 2025, and by August 2025, and a total of 180 patients have been recruited. The study is progressing according to plan, with recruitment anticipated to be completed by November 2025, aligning with the pre-specified timeline.

## Data Availability

De-identified datasets, code, and protocol will be available on reasonable request to the corresponding authors.

## Abbreviations

APS: Acute pain syndrome
CTRI: Clinical trial registry of India
ECOG: Eastern Cooperative Oncology Group
EORTC: European Organisation for Research and Treatment of Cancer
CIPN 20: Chemotherapy-induced peripheral neuropathy
FACT-B: Functional Assessment of Cancer Therapy—Breast
BPI-SF: Brief Pain Inventory- short form
CRF: Case Report Form
MAO: Monoamine oxidase inhibitors
NSAIDs: Nonsteroidal anti-inflammatory drugs
SSRI: Selective serotonin reuptake inhibitors
SAR: Serious adverse reactions
CTCAE: Common Terminology Criteria for Adverse Events
SAE: Serious adverse events
IEC: Institutional Ethics Committee
DCGI: Drug Controller General of India
DSMB: Data safety and monitoring board

## References

[1] Rodríguez-Antona C. Pharmacogenomics of paclitaxel. Pharmacogenomics. 2010;11(5):621–3. 20415548 10.2217/pgs.10.32

[2] Sati P, Sharma E, Dhyani P, Attri DC, Rana R, Kiyekbayeva L, et al. Paclitaxel and its semi-synthetic derivatives: comprehensive insights into chemical structure, mechanisms of action, and anticancer properties. Eur J Med Res. 2024;29(1):90. 38291541 10.1186/s40001-024-01657-2 PMC10826257

[3] Reeves BN, Dakhil SR, Sloan JA, Wolf SL, Burger KN, Kamal A, et al. Further data supporting that the paclitaxel-associated acute pain syndrome is associated with the development of peripheral neuropathy: NCCTG trial N08C1. Cancer. 2012;118(20):5171–8. 22415454 10.1002/cncr.27489 PMC3376686

[4] Loprinzi CL, Reeves BN, Dakhil SR, Sloan JA, Wolf SL, Burger KN, et al. Natural history of paclitaxel-associated acute pain syndrome: prospective cohort study NCCTG N08C1. J Clin Oncol. 2011;29(11):1472–8. 21383290 10.1200/JCO.2010.33.0308 PMC3082985

[5] Mols F, Beijers T, Vreugdenhil G, van de Poll-Franse L. Chemotherapy-induced peripheral neuropathy and its association with quality of life: a systematic review. Support Care Cancer. 2014;22(8):2261–9. 24789421 10.1007/s00520-014-2255-7

[6] Loprinzi CL, Maddocks-Christianson K, Wolf SL, Rao RD, Dyck PJB, Mantyh P, et al. The paclitaxel acute pain syndrome: sensitization of nociceptors as the putative mechanism. Cancer J. 2007;13(6):399–403. 18032978 10.1097/PPO.0b013e31815a999b

[7] Paclitaxel and its semi-synthetic derivatives: comprehensive insights into chemical structure, mechanisms of action, and anticancer properties | Eur J Med Res| Full Text. Available from: https://eurjmedres.biomedcentral.com/articles/10.1186/s40001-024-01657-2. Cited 2025 Aug 22. 10.1186/s40001-024-01657-2 PMC10826257 38291541

[8] Dougherty PM, Cata JP, Cordella JV, Burton A, Weng HR. Taxol-induced sensory disturbance is characterized by preferential impairment of myelinated fiber function in cancer patients. Pain. 2004;109(1–2):132–42. 15082135 10.1016/j.pain.2004.01.021

[9] Yoshida T, Sawa T, Ishiguro T, Horiba A, Minatoguchi S, Fujiwara H. The efficacy of prophylactic Shakuyaku-Kanzo-to for myalgia and arthralgia following carboplatin and paclitaxel combination chemotherapy for non-small cell lung cancer. Support Care Cancer Off J Multinatl Assoc Support Care Cancer. 2009;17(3):315–20. 10.1007/s00520-008-0508-z 18839222

[10] Saito Y, Kobayashi M, Yamada T, Sakakibara-Konishi J, Shinagawa N, Kinoshita I, et al. Efficacy of additional dexamethasone administration for the attenuation of paclitaxel-associated acute pain syndrome. Support Care Cancer. 2020;28(1):221–7. 31016422 10.1007/s00520-019-04808-y

[11] Zhang J, Gao HF, Yang C, Zhu T, Ji F, Yang M, et al. Prevention of taxane-associated acute pain syndrome with etoricoxib for patients with breast cancer: a phase II randomised trial. Eur J Cancer. 2022;171:150–60. 35724467 10.1016/j.ejca.2022.05.019

[12] Shinde SS, Seisler D, Soori G, Atherton PJ, Pachman DR, Lafky J, et al. Can pregabalin prevent paclitaxel-associated neuropathy?–an ACCRU pilot trial. Support Care Cancer Off J Multinatl Assoc Support Care Cancer. 2016;24(2):547–53. 10.1007/s00520-015-2807-5 26155765

[13] Nguyen VH, Lawrence HJ. Use of gabapentin in the prevention of taxane-induced arthralgias and myalgias. J Clin Oncol. 2004;22(9):1767–9. 15118009 10.1200/JCO.2004.99.298

[14] Jacobson SD, Loprinzi CL, Sloan JA, Wilke JL, Novotny PJ, Okuno SH, et al. Glutamine does not prevent paclitaxel-associated myalgias and arthralgias. J Support Oncol. 2003;1(4):274–8. 15334869

[15] Scholz BA, Hammonds CL, Boomershine CS. Duloxetine for the management of fibromyalgia syndrome. J Pain Res. 2009;2:99–108. 21197298 PMC3004624

[16] Frakes EP, Risser RC, Ball TD, Hochberg MC, Wohlreich MM. Duloxetine added to oral nonsteroidal anti-inflammatory drugs for treatment of knee pain due to osteoarthritis: results of a randomized, double-blind, placebo-controlled trial. Curr Med Res Opin. 2011;27(12):2361–72. 22017192 10.1185/03007995.2011.633502

[17] Ormseth MJ, Scholz BA, Boomershine CS. Duloxetine in the management of diabetic peripheral neuropathic pain. Patient Prefer Adherence. 2011;5:343–56. 21845034 10.2147/PPA.S16358 PMC3150163

[18] Meng J, Zhang Q, Yang C, Xiao L, Xue Z, Zhu J. Duloxetine, a balanced serotonin-norepinephrine reuptake inhibitor, improves painful chemotherapy-induced peripheral neuropathy by inhibiting activation of p38 MAPK and NF-κB. Front Pharmacol. 2019;10:365. 31024320 10.3389/fphar.2019.00365 PMC6465602

[19] Rodrigues-Amorim D, Olivares JM, Spuch C, Rivera-Baltanás T. A systematic review of efficacy, safety, and tolerability of duloxetine. Front Psychiatry. 2020;11:554899. 33192668 10.3389/fpsyt.2020.554899 PMC7644852

[20] Klein I, Lehmann HC. Pathomechanisms of paclitaxel-induced peripheral neuropathy. Toxics. 2021;9(10):229. 34678925 10.3390/toxics9100229 PMC8540213

